# Modelling study of dimerization in mammalian defensins

**DOI:** 10.1186/1471-2105-7-S5-S17

**Published:** 2006-12-18

**Authors:** Anita Suresh, Chandra Verma

**Affiliations:** 1 Biomolecular Modelling and Design Group, Bioinformatics Institute, 30 Biopolis Street, #07-01 Matrix, Singapore 138671

## Abstract

**Background:**

Defensins are antimicrobial peptides of innate immunity functioning by non-specific binding to anionic phospholipids in bacterial membranes. Their cationicity, amphipathicity and ability to oligomerize are considered key factors for their action. Based on structural information on human β-defensin 2, we examine homologous defensins from various mammalian species for conserved functional physico-chemical characteristics.

**Results:**

Based on homology greater than 40%, structural models of 8 homologs of HBD-2 were constructed. A conserved pattern of electrostatics and dynamics was observed across 6 of the examined defensins; models backed by energetics suggest that the defensins in these 6 organisms are characterized by dimerization-linked enhanced functional potentials. In contrast, dimerization is not energetically favoured in the sheep, goat and mouse defensins, suggesting that they function efficiently as monomers.

**Conclusion:**

β-defensin 2 from some mammals may work as monomers while those in others, including humans, work as oligomers. This could potentially be used to design human defensins that may be effective at lower concentrations and hence have therapeutic benefits.

## Background

Antimicrobial peptides (AMPs) are important components of the innate immunity of a wide range of organisms and present the first line of defence against invading microorganisms. Typically cationic, the peptides act against bacteria, fungi, and viruses through mechanisms involving membrane disruption or pore formation leading to leakage of cell content and destruction [1]. A major family of AMPs found in mammals, plants and insects is that of the defensins – small, cationic peptides containing one or more disulfide bridges. The human defensins are 30–45 amino acids in length, with three intramolecular disulfide bonds, and are classified into the α and β types based on the pattern of the disulfide bonds and mode of release. The α-defensins are involved in systemic and small intestinal host defence while the β-defensins protect mucosal epithelia [2]. The expression of most β-defensins, including the human β-defensin 2 (HBD-2) and the mouse β-defensin 3 is induced or upregulated upon microbial infection. The human β-defensins have potent antimicrobial activity and also attract T-lymphocytes and immature dendritic cells as part of an inflammatory response, thereby playing a key role in adaptive immunity [3].

It is postulated that cationicity, hydrophobicity as well as the ability to oligomerize are key determinants of the mode and intensity of action of antimicrobial peptides. The crystal structure of human α-defensin hNP3 revealed that it formed a dimer containing a six-stranded β-sheet region [4]. NMR studies indicate that HNP-1 can also form dimers or higher-order aggregates in solution [5]. The recent identification of Defr1, a covalently cross-linked mouse peptide of the defensin family gave weight to the idea that defensin antimicrobial activity is linked to its ability to form stable higher-order structures [6]. The observation of higher order structures of HBD3 by native gel electrophoresis allowed modelling of an HBD3 dimer mediated by noncovalent electrostatic interactions between residues on the second β-sheet [7]. Structure elucidation of HBD-2, the first human β-defensin structure determined, gave two crystallographic forms, an orthorhombic form comprising a dimer resulting from noncovalent interactions of the first β-sheet of each monomer (PDB: 1FD3, 1.35 Å), and a monoclinic di-octamer formed by amide backbone interactions within the crystallographic unit cell (PDB: 1FD4, 1.7 Å) [8]. The peptide is found in solution mainly as dimers although it is likely to form higher-order oligomers either in higher concentrations that are induced by pathogen attack, or in interactions with the lipid membranes of the bacterial cells.

Defensins are highly cationic – mammalian defensins carry charges ranging from +6 to +12, and yet they are relatively small – 25–45 amino acids long in the case of the mammalian mature peptides. In an attempt to understand how a protein so small and highly charged can overcome the gradient of charge-charge repulsions and aggregate, we have begun a series of detailed investigations. Using the dimer as the simplest model of oligomerization, we examine this feature from an evolutionary perspective and perform a comparative study of HBD2 and a series of homologous defensins from 8 other mammals (sequence identity greater than 40%). Based on a multiple sequence alignment of these 8 sequences against HBD-2, we construct structural models of the dimeric species and then examine the dynamic consequences of these structures through atomistic computer simulations, the energetics of these associations and their functional implications.

## Results and Discussion

**Figure 1:**
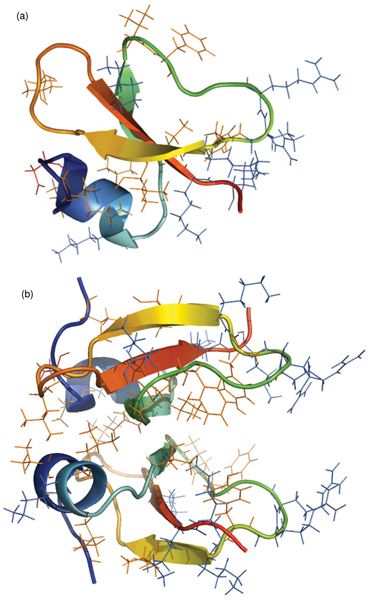
**Monomeric and dimeric representations of HBD-2**. The structure of HBD-2 monomer (a) and dimer (b), taken from the crystal structure 1FD3 [8] shown as cartoons in rainbow colouring from N- to C-terminal, with line representations of cationic residues in blue, hydrophobic residues in orange, and anionic residues in red.

The human HBD-2 possesses 7 cationic residues (2 Arg and 5 Lys, offset by one anionic Asp), resulting in a charge of +12 for the dimeric form. A histidine residue that is present is neutral in the conditions examined. The charged residues are distributed on the surface of the molecule except at the dimeric interface, which is comprised largely of hydrophobic residues. The dimer interface, which buries a surface area of ~818Å^2^, is formed between the first β-strands of two monomers, mainly via hydrogen bonds between the backbone atoms of Cys15, and aided by van der Waals contacts made by the residues Pro5, Ala13, Ile14, Cys15, His16, and Pro17 [8]. Figure 1 shows the structure of the monomeric and dimeric structures of HBD-2, coloured by polarity. The separation of the charged sidechains, away from the dimerization interface is clear. This suggests at first glance that dimerization is driven largely by hydrophobic forces and little or no desolvation penalties are incurred for burying any charges.

Multiple sequence alignment of HBD-2 and its 8 homologous sequences (Table 1) reflects the evolutionary clustering of the parent species, with distinct patterns of amino acid distributions between primates, the trio of pig-rat-cow and the remaining mouse-sheep-goat. The rat and mouse homologs each have one residue less than the remaining sequences, corresponding to positions within the long loop between the first and second β-strands (refer to Figure 1). While the mature C-terminal ends are all positively charged due to the presence of excess arginines and lysines, HBD-2 and its primate homologs lack the additional terminal cationic residue, seen among the rest of the organisms.

**Figure 2:**
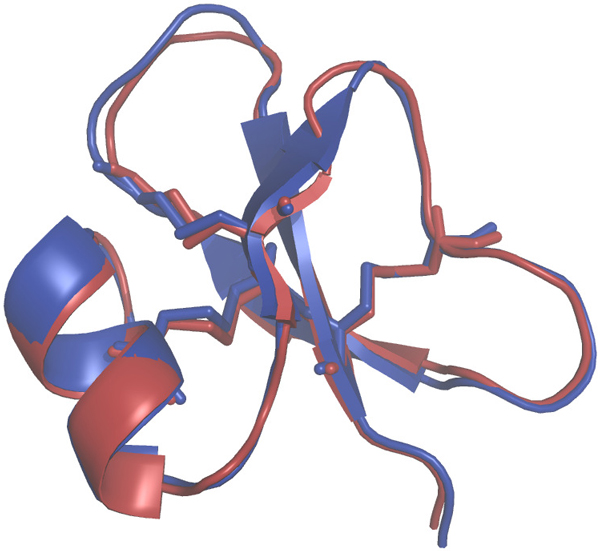
**Comparison of homology models.**  Superposition of 3-dimensional homology models of pig defensin (Swiss-Prot: BD01_PIG) constructed using Modeller (in blue) and using SDPMOD (in red), revealing the overall similarity in backbone conformations.

To gain structural insights into the spatial dispositions of these residues, we have used these alignments to construct 3-dimensional atomistic models of the dimeric forms of the 8 homologs using the program Modeller (see Methods). Further, to evaluate the accuracy of the employed modelling protocol, we compared the monomeric form of one of the models (pig homolog) with the corresponding model generated using a program that specializes in generating structural models based on homology of small disulfide-bonded proteins, the SDPMOD server [9]. The two models superposed with a backbone root mean squared deviation of only 0.6 Å (see Figure 2) with any significant differences confined to the loop regions. This suggests that the methodology we employed was robust. Our choice of Modeller was driven by the need to build structural models of dimers.

**Figure 3:**
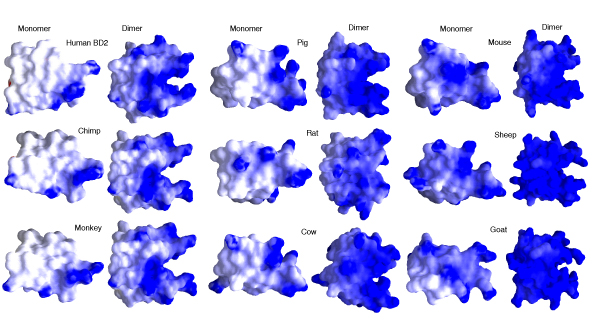
**Electrostatic surfaces of defensin monomers and dimers.**  Electrostatic potentials, computed using GRASP [37] mapped on to the molecular surfaces of defensin monomers and dimers for each homolog (the potentials range from -10 *kT/e *for red to +10 *kT/e *for blue).

As expected from the alignment and the similarity of the distribution of the cationic residues (Table 1), the overall electrostatic potentials mapped on to the molecular surfaces (Figure 3) look similar in spatial disposition and yet reflect the increasing cationicity across the species. A distribution into three classes, reflecting that seen in the sequences, is evident: human-chimp-monkey; pig-rat-cow and mouse-sheep-goat. Upon dimerization, this property is seen to increase synergistically (Figure 3). What is intriguing is that this enhancement of cationicity is not as strong amongst the mouse, sheep and goat β-defensins. This perhaps arises from the monomers being rich in cationic residues in these 3 cases (+10, +12, +12 for mouse, sheep and goat respectively), leading to a strong positive potential that envelops the whole surface. Thus, the significant increase seen upon dimerization in the other species is not seen in these 3 species because the monomeric systems in these 3 species are already highly charged. The increase across the species does suggest that dimerization will lead to a stronger charge-charge interaction with the negatively charged bacterial membrane in contrast to the membrane-monomer interactions. There is as yet no clear understanding of how the interplay of electrostatic and hydrophobic forces leads to oligomerization, even though it is known to be important [10,11]. Rousseau [12] pointed out that arginine and lysine residues function to oppose aggregation by a combination of their charges, which are repulsive in closely packed complexes, and their long, flexible side-chains, which are entropically unfavourable. However, HBD-2, which did not show a propensity to aggregate based on pure hydrophobic interactions (data not shown), does carry a large number of hydrophobic residues and there are no cationic residues at the dimerization interface. The hydrophobic residues are well distributed throughout the sequence and interspersed with several polar residues; this could theoretically reduce the tendency of β-aggregation. Experimentally however, dimers are seen and this underscores again the fine balance that coexists between opposing forces. The dimer interface comprises a few hydrophobic residues and also allows for backbone hydrogen bonds, e.g. between Cys15 in HBD-2 dimer, seen in 1FD4 [8].

**Figure 4:**
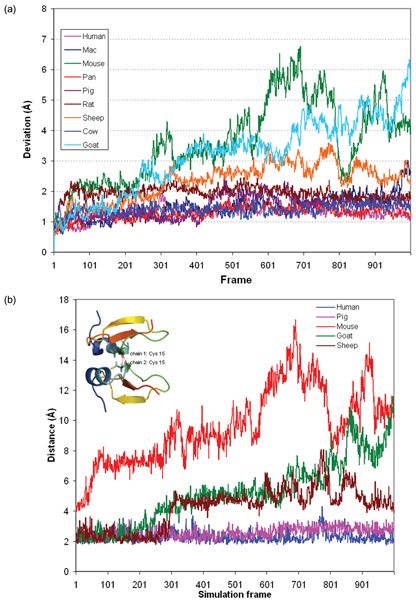
**Atomic changes in defensin dimers**. (a) Root mean squared deviations of the Cα atoms of defensin dimers from their starting modelled structures over 10 ns of MD simulations; (b) Distance between chains in human, pig, mouse, sheep and goat defensin dimers, monitored as the distance between the centroids of the interfacial Cys residues over 10 ns MD simulations showing the drift arising from repulsive interactions in mouse, goat and sheep. The corresponding distances for other species not shown here follow the stable trend seen for human and pig dimers.

**Figure 5:**
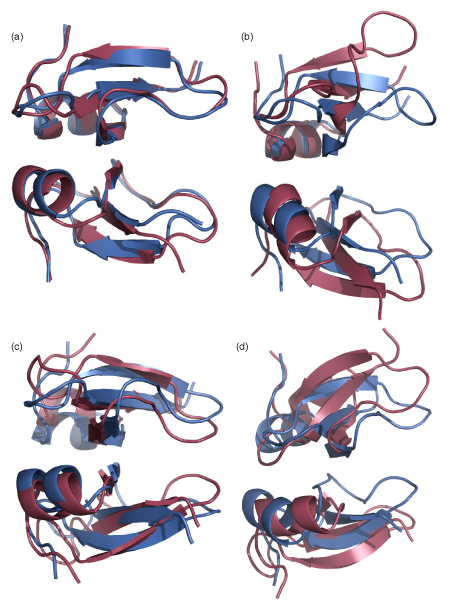
**Defensin dimer conformations during MD simulations.**  Superposition of the structures of defensin dimers from (a) Human (b) Mouse (c) Sheep (d) Goat highlighting the drift between the monomers arising from charge-charge repulsions during the MD simulations; the first (blue) and the last (pink) frames from the 10 ns simulations are shown.

**Figure 6:**
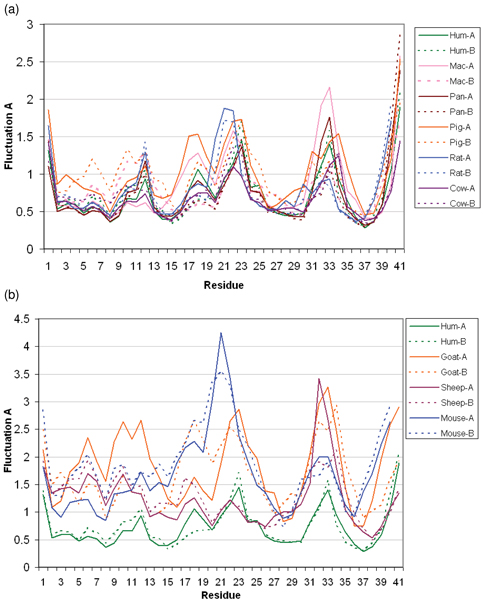
**Atomic fluctuations of defensin dimers**. RMSF plots for individual chains (A and B) of defensin dimers from (a) Human, Mac (Monkey), Pan (Chimp), Pig, Rat, Cow (b) Human, Goat, Sheep, Mouse, as computed for Cα atoms over 10 ns MD simulations. The corresponding secondary structure schema is shown below each of the graphs.

We subsequently carried out molecular dynamics simulations on each of the dimers. The drifts from the initial structures plateau for six dimers while those from mouse, sheep, and goat show large increases (Figure 4a). While there is no direct correlation between the amount of positive charge and the extent of deviation, we observe differences in the behaviours of these three relative to that of the others. We note that the distance between the dimers, as measured by monitoring the separation between the centroids of the interfacial Cys residues shows stable separations for the dimers except for the three outliers namely mouse, sheep and goat (Figure 4b; data has been shown for the three outliers and for human and pig; the other species essentially follow the stable pattern seen in human and pig and hence have been omitted for clarity of the figure). Indeed visual inspections show that the monomers in these three cases drift away from each other by the end of the simulations (Figure 5) – a clear indication that charge-charge repulsions dominate. Examination of the average fluctuations over the simulations reveals that the fluctuations in mouse, sheep and goat are much larger than in the other species – once again reflective of higher motions arising from the dominating intramolecular repulsions (Figure 6a, 6b).

**Figure 7:**
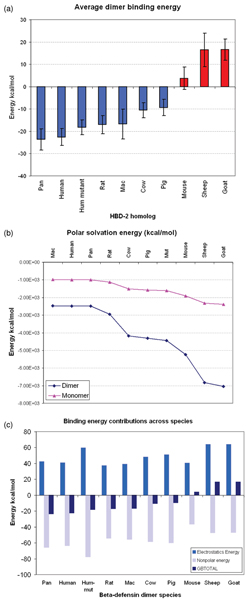
**Energetics of defensin dimers**.  (a) Total computed average binding energy of defensin dimers (b) Solvation energy of monomers and dimers of defensin homologs (c) Polar and nonpolar contributions to binding energy of dimers.

The average dimerization energies, computed across the 10 ns simulations for each dimer, are strongly stabilizing of the dimeric forms except in the three outliers (Figure 7a). As expected for such a highly charged system, the solvation energies of the monomers are favourable; the fact that the solvation of the dimers witnesses a synergistic stabilization shows that the charges are not buried upon dimerization (Figure 7b). However, the Coulombic repulsion far outweighs the gain in energy from solvation (Figure 7c) and it is the hydrophobic energy of dimerization that really stabilizes the dimers; however, this is not the case for the outliers, where the overall binding energy, dominated by Coulombic repulsions, does not favour dimerization.

**Figure 8:**
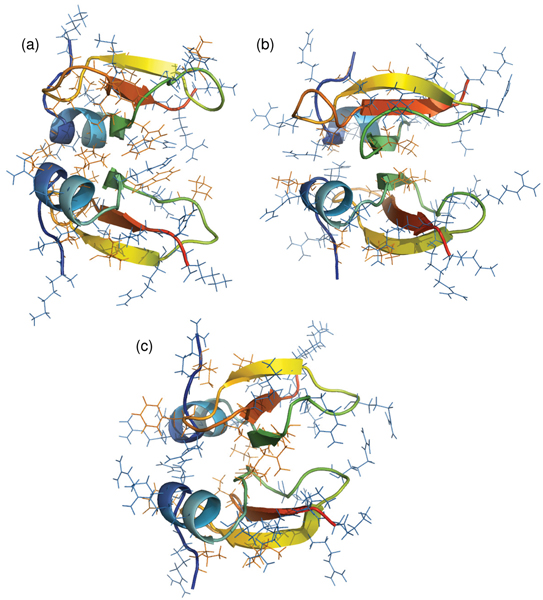
**Polar nature of defensin dimers**. Distribution of cationic and hydrophobic residues in dimeric defensin homologs of (a) Mouse (Swiss-Prot: BD03_MOUSE), (b) Sheep (BD02_SHEEP) and (c) Goat (BD02_CAPHI).  The structures are represented as cartoons in rainbow colouring from N- to C-terminal, with line representations of cationic residues in blue and hydrophobic residues in orange.

We see that the three outliers are characterized structurally by a higher density of cationic residues in the immediate vicinity of the putative dimerization interface (Figure 8) when compared to HBD-2 (Figure 1). This would lead to the large repulsions we see above disfavouring dimerization and to the observed drift in the simulations of the monomers away from each other.

Cationicity is typically correlated with antimicrobial activity, and dimerization or oligomerization brings about a synergistic increase in cationicity of the peptide complex over that of the monomer. This oligomerization may indeed be an innate property as seen in these models, or it may be brought about by an increase in local concentrations that result as a response to some environmental conditions, such as the presence of other molecules [13]. However, in the case of the defensin homologs of mouse, sheep and goat, their high net charge may obviate the need for dimerization for the purpose of increased charge and hence activity. It is possible that the HBD-2 homologs of these organisms do not form dimers and instead act in the monomeric form against bacterial membranes. The higher charges of certain defensins, such as the +12 net charge of goat or sheep defensins, may reflect the pathogen environment of the species. A larger cationicity may indicate the potential to combat a wider variety of bacteria. Indeed, HBD-3 is the most cationic peptide among the known human β-defensins. It is also the most potent human β-defensin and is highly versatile in its pathogen killing ability, acting against Gram-negative and Gram-positive bacteria and fungi [7]. While cationicity alone is not sufficient for antimicrobial effect, it is crucial for the initial recognition and docking of the defensins to the microbial membrane. The charge distribution variance among the examined species may be indirectly related to the environmental pathogens each species is typically exposed to in its lifetime, knowledge of which will be useful in the understanding and design of improved defensin derivatives.

### Mutating HBD-2 to increase cationicity

**Figure 9:**
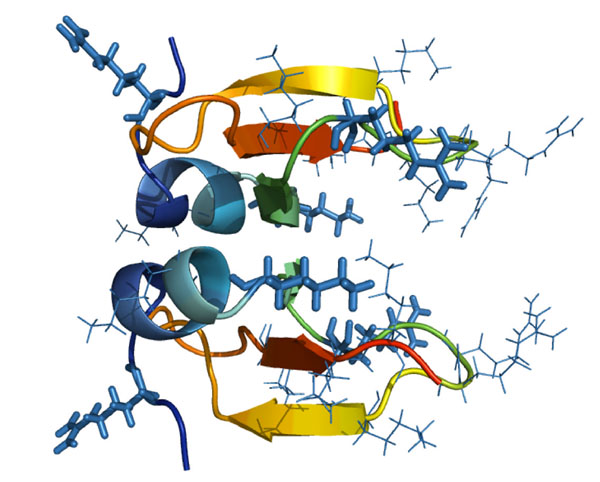
**Structural model of mutant HBD-2 (Hum-mut)**. The chains are each displayed in rainbow colouring from N- to C-terminus. All cationic residues are shown in blue, and the positions of the Arg mutations are highlighted in stick representation.

To test the above hypothesis, we carried out *in silico *mutation of HBD-2 to increase the overall cationicity. Based on the multiple sequence alignment, neutral residues in three positions within the human model structure were replaced with Arg (Figure 9), guided by the presence of homologous Arg in the corresponding positions in goat and sheep; this resulted in an overall charge of +18 for this mutant HBD-2. The computed dimerization energies showed that the mutant dimer was 2% less stable than the wild type dimer. The introduction of the excess positive charges leads to destabilized electrostatics (~50% more than in the wild type dimer); this was offset by an almost equal contribution from hydrophobic forces. This again underscores the tenuous links between opposing forces and structural dispositions of charges in proteins. But the observation that our mutations destabilized the dimeric species provides us with confidence that we are proceeding in the right direction for the design of suitable analogues.

### Towards a model for peptide-membrane interaction

So how do defensins (and antimicrobial peptides in general) disrupt bacterial membranes? Clearly the process involves recognition, assembly and association (in some order), followed by one or more mechanisms whereby the defensin disrupts the regular structure and function of the membrane [1]. One suggestion was that the membrane interaction was independent of the peptide's secondary structure and involved the overall spatial arrangement of polar and hydrophobic residues [14]. Further, given the amphipathic nature of membranes, the initial steps of membrane association and the final alignment and localization of the peptides within membranes would also depend on some combination of hydrophobic and electrostatic interactions [15]. This leads to one of the more commonly accepted models, the carpet model, which postulates that the peptides, at sufficiently high concentrations, aggregate into micelles and disrupt the membrane [16]. While direct evidence of peptide aggregation in the membrane has been lacking, a solid-state NMR study yielded a model of protegrin-1 aggregation in lipid bilayers [17]. Thus, it is possible that higher order oligomers of HBD-2 transiently form upon association with the membrane, although such a phenomenon is yet to be observed through biophysical methods.

**Figure 10:**
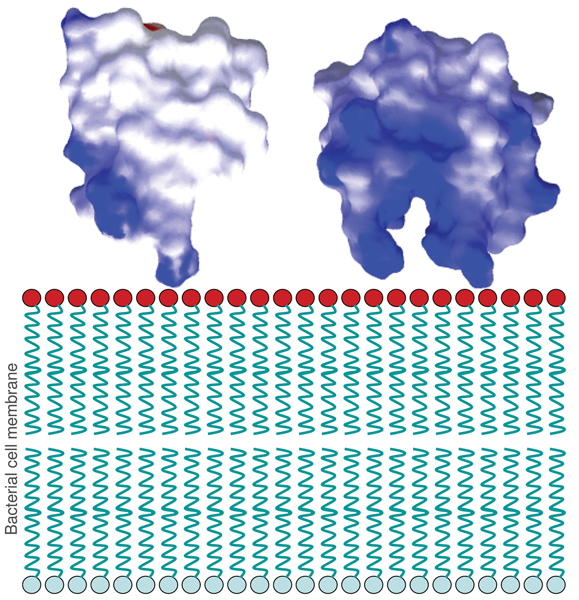
**Cationic claw of defensin dimers**. The HBD-2 structure (from 1FD3) possesses a highly cationic "tail" region, and upon dimerization, the pair of tails project from the same end of the dimer, resulting in a claw-like appearance.

It is known that unstructured clusters of basic residues on proteins can produce strong localized electrostatic potentials, which can enhance their attraction to anionic membranes [18]. These lead to accumulation/lateral organization of the proteins at the membrane surfaces [19] and can mediate the self-promoted uptake of defensins across the cell wall followed by interaction with the anionic bacterial cytoplasmic membrane [20]. The HBD-2 structure possesses a highly positively-charged "tail" region. Upon dimerization, the tails of both monomers project from the same end of the dimer, resulting in a claw-like appearance (Figure 10). It is possible that the monomer or even the dimer employs this projecting cationic region to make initial contact with, or even a prehensile grasp of, the outer edge of the bacterial membrane. This could be followed by reorientations of the HBD-2 molecules, possibly in association with other HBD-2 molecules, to allow the hydrophobic residues to penetrate the lipid bilayer. The multiple sequence alignment of HBD-2 and related β-defensin sequences (Table 1) shows a conservation of cationic residues in this tail region. Although the actual number of these cationic residues varies between species and type of defensin, it does point towards the possibility of such an occurrence.

This is the very first study to our knowledge which has looked at the propensity of defensins to oligomerize and the concomitant functional relevance. While there is very little experimental data to date on the kinetics or energetics of such processes in defensins, a better understanding of the mechanisms underlying the broad-spectrum bacterial killing by antimicrobial peptides remains of paramount interest. In view of the growing resistance of pathogens to conventional antibiotics, this would help accelerate developments of new potent antibiotics. We hope that this kind of study will lead to further and more detailed experimental and theoretical investigations on the mode of action of antimicrobial peptides.

## Conclusion

We have used the dimer as the simplest model of oligomerization for a comparative study of the dimerization and relative cationicity of defensin HBD-2 and modelled homologs from 8 other mammals. A clear clustering of overall cationicity and potential to oligomerize was seen. While the defensins from 6 species showed a propensity to dimerize, the dimerization potentials of those from mouse, sheep and goat tend to disfavour dimeric assemblies. This suggests that either these outlier defensins act in the monomeric form against bacterial membranes or that they function as much higher oligomeric species; the high charge densities tend to suggest that their monomeric forms are the functional units.

## Methods

Using the mature peptide sequence of HBD-2 as a query in Blastp [21], several hits across species were obtained. The top 20 hits were aligned against the query sequence in a multiple sequence alignment using ClustalX [22]. We chose the top sequences (≥ 40% sequence identity), originating from 8 unique species for further study.

Monomer and dimer models of eight homologous mammalian sequences of HBD-2 were built using MODELLER [23] based on the structure of a dimeric complex of HBD-2 (chains C and D of PDB: 1FD3, X-ray crystal structure resolved to 1.35 Å) [8]. The side-chains were built and optimized using SCWRL [24]. Structural representations were visualized using Pymol [25] and simulations were viewed using VMD [26].

The solvation energies of the monomeric and dimeric forms of each defensin species examined was calculated by solving the non-linearized form of the PB equation in parallel with the Adaptive Poisson-Boltzmann Solver (APBS) [27].

Molecular dynamics (MD) simulations were performed using the AMBER 8.0 package and parm99 force field [28]. The initial structures were solvated with three- point transferable intermolecular potential (TIP3P) water molecules [29] and appropriate number of Chloride ions in a rectangular box to neutralize the system; the box dimensions ensured that any protein atom was at least 8 Å away from the wall of the box. After energy minimization, MD simulations were performed for 130 ps at constant temperature (300 K) and pressure (1 atm) with periodic boundary conditions, particle-mesh Ewald summation, and a 1-fs time step to heat and equilibrate the system. This was followed by production runs of 10 ns duration for each simulation. Structures were saved every 10 ps for analysis. The relative binding energies for dimer formation were computed using the MM-PBSA module of AMBER 8.0, employing molecular mechanics and a continuum solvent model [30]. This method calculates a gas-phase contribution to binding using an all-atom force field and incorporates the influence of solvent via the Generalized Born (GB) solvent models [31,32].

## Authors' contributions

AS carried out the sequence alignment, molecular modeling and simulations, and drafted the manuscript. AS and CV conceived of the study. CV supervised the study design and coordination and edited the manuscript. Both authors read and approved the final manuscript.

## Figures and Tables

**Table 1:** Multiple Sequence alignment of mammalian homologs of HBD-2. The chosen sequences from SWISSPROT have ≥ 40% sequence identity to human defensin HBD-2; Sequence details (Swiss-Prot id:*organism*): BD02_HUMAN: *Homo sapiens *(Hum); BD02_PANTR: *Pan troglodytes *(Chimp); BD02_MACMU: *Macaca mulatta *(Monkey); BD04_RAT: *Rattus norvegicus *(Rat); BD03_MOUSE: *Mus musculus*; BD01_PIG: *Sus scrofa *(Pig); TAP_BOVIN: *Bos taurus *(Cow); BD01_SHEEP: *Ovis aries *(Sheep); BD01_CAPHI: *Capra hircus *(Goat). The net charge for each sequence is shown in the last column. Cationic residues are shown in italics, conserved positions are indicated with "*" if they are invariant and ":" if they are of similar polarity.

Human	G	I	G	D	P	V	T	**C**	L	*K*	S	G	A	I	**C**	H	P	V	F	**C**	P	*R*	*R*	Y	*K*	**Q**	**I**	**G**	T	**C**	G	L	P	G	T	*K*	**C**	**C**	*K*	*K*	P	+6
Chimp	G	I	S	D	P	V	T	**C**	L	*K*	S	G	A	I	**C**	H	P	V	F	**C**	P	*R*	*R*	Y	*K*	**Q**	**I**	**G**	T	**C**	G	L	P	G	T	*K*	**C**	**C**	*K*	*K*	P	+6
Monkey	G	I	G	D	P	V	T	**C**	L	*K*	N	G	A	I	**C**	H	P	V	F	**C**	P	*R*	*R*	Y	*K*	**Q**	**I**	**G**	T	**C**	G	L	P	G	T	*K*	**C**	**C**	*K*	*K*	P	+6
Pig	N	I	G	N	S	V	S	**C**	L	*R*	N	*K*	G	V	**C**	M	P	G	*K*	**C**	A	P	*K*	M	*K*	**Q**	**I**	**G**	T	**C**	G	M	P	Q	V	*K*	**C**	**C**	*K*	*R*	*K*	+9
Cow	G	V	G	N	P	V	S	**C**	V	*R*	N	*K*	G	I	**C**	V	P	I	*R*	**C**	P	G	S	M	*K*	**Q**	**I**	**G**	T	**C**	V	G	*R*	A	V	*K*	**C**	**C**	*R*	*K*	*K*	+9
Sheep	G	V	*R*	N	*R*	L	S	**C**	H	*R*	N	*K*	G	V	**C**	V	P	S	*R*	**C**	P	*R*	H	M	*R*	**Q**	**I**	**G**	T	**C**	*R*	G	P	P	V	*K*	**C**	**C**	*R*	*K*	*K*	+12
Goat	G	I	*R*	S	*R*	*R*	S	**C**	H	*R*	N	*K*	G	V	**C**	A	L	T	*R*	**C**	P	*R*	N	M	*R*	**Q**	**I**	**G**	T	**C**	F	G	P	P	V	*K*	**C**	**C**	*R*	*K*	*K*	+12
Rat	S	I	N	N	P	I	T	**C**	L	T	*K*	G	G	V	**C**	W	G	-	P	**C**	T	G	G	F	*R*	**Q**	**I**	**G**	T	**C**	G	L	P	*R*	V	*R*	**C**	**C**	*K*	*K*	*K*	+7
Mouse	*K*	I	N	N	P	V	S	**C**	L	*R*	*K*	G	G	*R*	**C**	w	N	-	*R*	**C**	I	G	N	T	*R*	**Q**	**I**	**G**	S	**C**	G	V	P	F	L	*K*	**C**	**C**	*K*	*R*	*K*	+10
																																										
		:		.			:	*			.		.		*					*					:	*	*	*	:	*						:	*	*	:	:		Charge
